# Protocol for the Cultural Translation and Adaptation of the World Endometriosis Research Foundation Endometriosis Phenome and Biobanking Harmonization Project Endometriosis Participant Questionnaire (EPHect)

**DOI:** 10.3389/fgwh.2021.644609

**Published:** 2021-03-26

**Authors:** Cise Mis, Gokcen Kofali, Bethan Swift, Pinar Yalcin Bahat, Gamze Senocak, Bahar Taneri, Lone Hummelshoj, Stacey A. Missmer, Christian M. Becker, Krina T. Zondervan, Bahar Yuksel Ozgor, Engin Oral, Umit Inceboz, Mevhibe B. Hocaoglu, Nilufer Rahmioglu

**Affiliations:** ^1^Faculty of Communication and Media Studies, Eastern Mediterranean University, Famagusta, Cyprus; ^2^Cyprus Women's Health Research Society (CoHERS), Nicosia, Cyprus; ^3^Wellcome Centre for Human Genetics, University of Oxford, Oxford, United Kingdom; ^4^Nuffield Department of Women's and Reproductive Health, Oxford Endometriosis CaRe Centre, University of Oxford, Oxford, United Kingdom; ^5^Department of Obstetrics and Gynecology, Health Science University, Istanbul Kanuni Sultan Suleyman Training and Research Hospital, Istanbul, Turkey; ^6^Department of Obstetrics and Gynaecology, Erzurum Ataturk University, Erzurum, Turkey; ^7^Department of Biological Sciences, Faculty of Arts and Sciences, Eastern Mediterranean University, Famagusta, Cyprus; ^8^Department of Genetics and Cell Biology, Faculty of Health, Medicine & Life Sciences, Institute for Public Health Genomics, Maastricht University, Maastricht, Netherlands; ^9^World Endometriosis Research Foundation (WERF), London, United Kingdom; ^10^Department of Epidemiology, Harvard T. H. Chan School of Public Health, Boston, MA, United States; ^11^Department of Obstetrics, Gynecology and Reproductive Biology, College of Human Medicine, Michigan State University, Grand Rapids, MI, United States; ^12^Esenler Maternity and Children's Hospital, Istanbul, Turkey; ^13^Department of Obstetrics and Gynecology, Bezmialem Vakif University, Istanbul, Turkey; ^14^Irenbe Women's Healthcare Centre, Izmir, Turkey; ^15^Department of Palliative Care, Policy and Rehabilitation, Cicely Saunders Institute, King's College London, London, United Kingdom; ^16^Department of Psychology, Faculty of Medicine and Faculty of Arts and Sciences, Eastern Mediterranean University, Famagusta, Cyprus

**Keywords:** endometriosis, standardization, harmonization, Turkish, questionnaire, EPHect, cross-cultural adaptation

## Abstract

Endometriosis affects 10% of women worldwide and is one of the most common causes of chronic pelvic pain and infertility. However, causal mechanisms of this disease remain unknown due to its heterogeneous presentation. In order to successfully study its phenotypic variation, large sample sizes are needed. Pooling of data across sites is not always feasible given the large variation in the complexity and quality of the data collected. The World Endometriosis Research Foundation (WERF) Endometriosis Phenome and Biobanking Harmonization Project (EPHect) have developed an endometriosis participant questionnaire (EPQ) to harmonize non-surgical clinical participant characteristic data relevant to endometriosis research, allowing for large-scale collaborations in English-speaking populations. Although the WERF EPHect EPQs have been translated into different languages, no study has examined the cross-cultural translation and adaptation for content and face validity. In order to investigate this, we followed the standard guidelines for cross-cultural adaptation and translation of the minimum version of the EPQ (EPQ-M) using 40 patients who underwent laparoscopic surgery in Turkey and 40 women in Northern Cyprus, aged between 18 and 55. We assessed the consistency by using cognitive testing and found the EPHect EPQ-M to be comprehensive, informative, and feasible in these two Turkish-speaking populations. The translated and adapted questionnaire was found to be epidemiologically robust, taking around 30–60 min to complete; furthermore, participants reported a similar understanding of the questions, showing that common perspectives were explored. Results from the cognitive testing process led to minor additions to some items such as further descriptive and/or visuals in order to clarify medical terminology. This paper illustrates the first successful cross-cultural translation and adaptation of the EPHect EPQ-M and should act as a tool to allow for further studies that wish to use this questionnaire in different languages. Standardized tools like this should be adopted by researchers worldwide to facilitate collaboration and aid in the design and conduction of global studies to ultimately help those affected by endometriosis and its associated symptoms.

## Introduction

Endometriosis is a chronic inflammatory condition whereby tissue that resembles the endometrium is found at sites outside of the uterus, including the pelvis, bladder, bowel, and ovaries (1). It affects around 10% of reproductive age women worldwide (~176 million women) and is one of the most common causes of pelvic pain and infertility (2). Endometriosis is heterogenous in presentation, with a wide variety of clinical presentations and behaviors, which means its causal mechanisms have been, so far, difficult to elucidate (1). The heterogeneity of the disease means that both its diagnosis and treatment are challenging as reliable diagnosis requires laparoscopic surgery. However, endometriomas and deep endometriosis can be visualized using ultrasonography or magnetic resonance imaging (MRI) (3, 4). Treatment options include removal or destruction of the disease tissue via laparoscopy or hormonal treatment and analgesics, which have unwanted side effects, high recurrence rates, and limited long-term support (1).

In order to successfully study the phenotypic variation seen in endometriosis, large sample sizes are needed. Given the large variation in the complexity and quality of data collection, pooling of data across study sites is not always feasible. The World Endometriosis Research Foundation (WERF) Endometriosis Phenome and Biobanking Harmonization Project (EPHect) has developed tools in order to facilitate the design and interpretation of collaborative studies, enabling large-scale, and epidemiologically robust research into endometriosis causes, diagnostic methods, and treatment improvements (5).

The WERF EPHect Working Group developed an endometriosis participant questionnaire (EPQ) (standard [EPect EPQ-S] and minimum [EPHect EPQ-M] versions) to evaluate non-surgical clinical participant characteristic data relevant to endometriosis. Although the WERF EPHect EPQs have been translated into different languages (endometriosisfoundation.org/ephect/2), no study has examined the cross-cultural translation and adaptation of the EPQ-M. Cross-cultural translation and adaptation is essential for content and face validity of the questionnaire in different languages. Using two Turkish-speaking populations to illustrate, we aim to provide a protocol to enable others to successfully implement and utilize the WERF EPQ-M, thus building upon the collaborative global effort in endometriosis research.

## Materials and Methods

### Study Sample

A total of 80 women were recruited into the study between December 2017 and September 2019 with the following inclusion criteria: aged between 18 and 50, no known cognitive impairment, and the ability to give consent. All recruitment and data collection were undertaken by experienced recruitment teams. The study population was recruited from three different sites including two hospital settings, namely, the Cerrahpasa University Medical Faculty in Istanbul and Ataturk University Medical Faculty Hospital in Erzurum, Turkey. The clinical sample included 40 women who were undergoing laparoscopic surgery in Turkey, and the general population sample consisted of 40 randomly selected women from households in Northern Cyprus.

### Study Design

**Figure 1:**
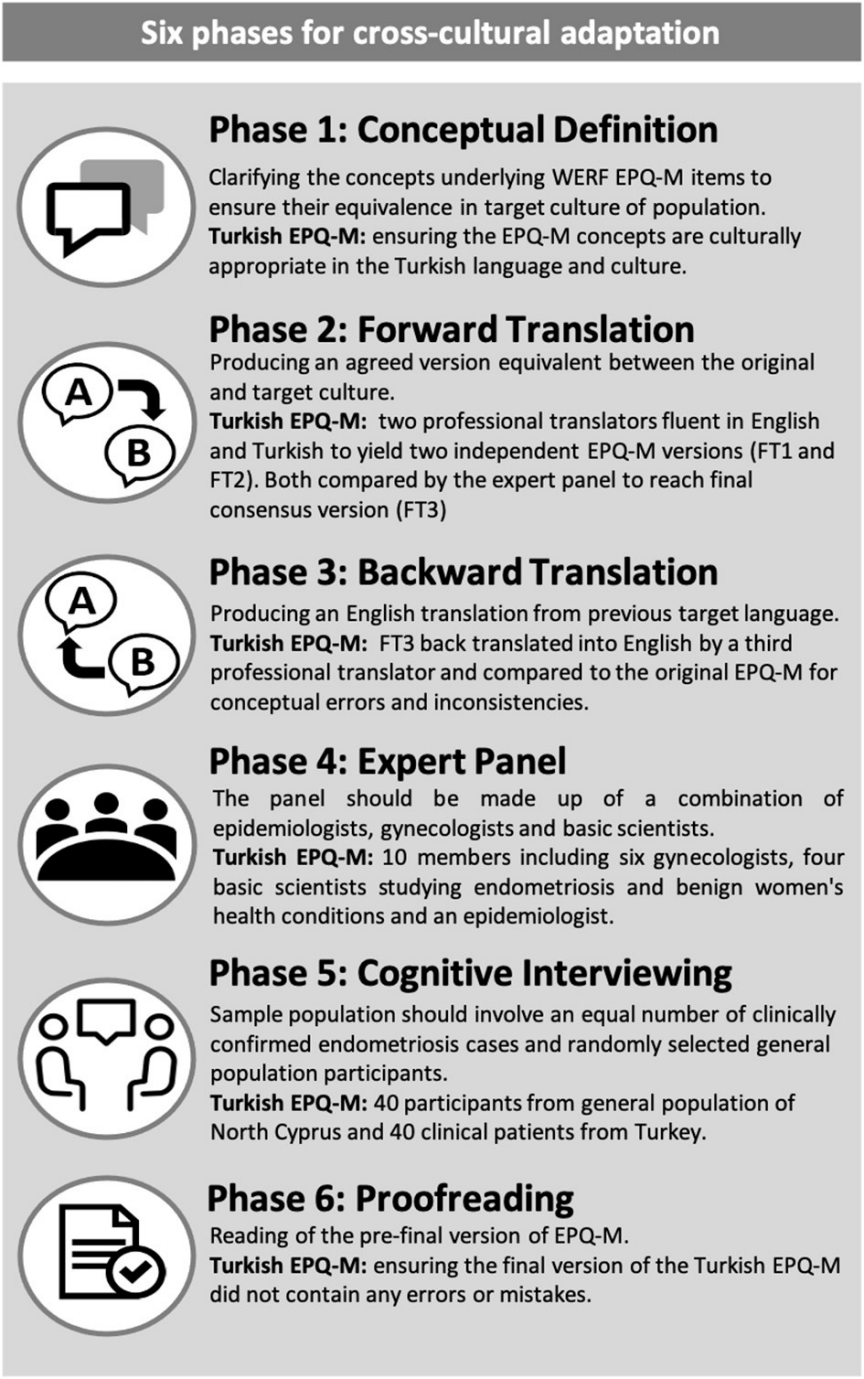
Overview of the translation and cultural adaptation process.

The cross-cultural translation and adaptation of the WERF EPHect EPQ-M was carried out using the recommended guidelines (6) and to COSMIN standards (7). The process was completed as follows: (1) conceptual equivalence, (2) forward translation, (3) backward translation, (4) expert panel revision, (5) cognitive testing, and (6) proofreading (Figure 1).

1. **Conceptual Equivalence**
  This stage involved completing a literature review relating to endometriosis and health and well-being related concepts in the target language database. Gynecologists were consulted to ensure that all concepts and questions in the EPHect EPQ-M were well-defined and relevant as well as to allow for any modifications of potentially culturally insensitive/unsuitable differences. In our study, we used two gynecologists to complete this process. Semantic equivalences were sought prior to this and specifically focused on key concepts and their definitions in Turkish.
2. **Forward Translation**
  The forward translation was carried out by two independent professional translators who were native speakers of both the target language and English. This process produced two independent versions of the EPHect EPQ-M in the target language (FT1 and FT2), and these versions were compared by an expert panel for differences to reach a final consensus and a final forward translation version (FT3). The expert panel comprised of epidemiologists, basic research scientists, and gynecologists (some with endometriosis expertise). For the Turkish version of the EPHect EPQ-M, our translators were located in Famagusta (Cyprus) and Istanbul (Turkey). The expert panel consisted of one epidemiologist/basic research scientist (also a member of the WERF EPHect working group), six gynecologists (five with endometriosis expertise and one general gynecologist), and three basic research scientists.
3. **Backward Translation**
  The FT3 was then translated into English by a third independent professional translator. The expert panel compared the back-translated English version with the original English version of the WERF EPHect EPQ-M for conceptual errors and inconsistencies. This step is important in ensuring that the FT3 is suitably consistent with the original version. The third independent professional translator in our study was based in Famagusta (Cyprus).
4. **Expert Panel Revision**
  Here, the expert panel reviewed all forward and back translations in order to reach consensus and arrive at a pre-testing version of the EPHect EPQ-M.
5. **Cognitive Testing**
  Cognitive testing is an evidence-based methodology designed to evaluate how questions are understood and interpreted by the target population (8). The research assistants sat opposite to the participants, and the participants were asked to read the instructions, questions, and answer options of the pre-testing EPHect EPQ-M. The participants were given the opportunity to indicate if there was anything on the questionnaire that was unclear to them, and these were then reviewed by the expert panel to arrive at the final version. The outcome of this process was the final Turkish WERF EPHect EPQ-M.
6. **Proofreading**
  The final EPQ-M was proofread to ensure that it did not contain any errors or mistakes.

### Ethical Approval

The COHERE Initiative was approved by the Oxford Tropical Research Ethics Committee (OxTREC) of the University of Oxford (OxTREC reference: 37-17) and the Ethics Committee of Eastern Mediterranean University (ETK00-2017-0240). The Turkish Endometriosis Genomic Study received ethics approval from the Cerrahpasa University Faculty of Medicine Ethics Group (52825153-604.01-01-20831). Informed consent was obtained from all participants.

## Results

### Conceptual Evidence

Both a review of published articles in the Turkish database and the expert review suggested that all concepts within the Turkish EPHect EPQ-M sufficiently addressed endometriosis-related symptomatology, history, and related reproductive health and lifestyle factors in the Turkish-speaking population. The question “How would you describe your ethnic origin?” was considered too intrusive by the participants in Turkey, and although it was deemed acceptable in the Turkish Cypriot community, it was excluded in this instance and left as an optional question for future studies given the potential sensitivities around the topic.

### Translations and Revisions

All of the main concepts (such as “menstrual period,” “pregnancy,” “fertility,” and “pelvic pain”) were found to have the same meaning in Turkish as they did on the original English EPHect EPQ-M and were therefore said to have sematic equivalence. Sematic equivalence was also evidenced in the back-translation process when the Turkish EPQ-M was translated back into English.

The main challenges in the forward translation process were surrounding the linguistic typology differences between Turkish and English. The Turkish language follows a subject–object–verb (SOV) sentence structure, whereas English follows a subject–verb–object (SVO) sentence structure. The backwards translation phase produced very similar translations; the only differences were caused by variations in the sentence structure and where the back translation produced a more indirect translation, e.g., “emergency pregnancy control” instead of “emergency contraception.”

Once the expert review panel had arrived at the pre-testing version of the EPHect EPQ-M, the questions were shortened to make the wording more succinct using the best colloquial terminology in Turkish reproductive health. Once consensus had been reached, the pre-testing version was taken forward into the cognitive testing phase.

### Cognitive Testing

A total of 80 women between the ages of 18 and 55 took part in the cognitive testing process (Table 1) with duration of questionnaire completion ranging from 30 to 60 min. Ninety-five percent of the participants self-reported to have a high-school degree or above in Northern Cyprus, Istanbul, and Turkey, whereas 60% of the participants based in the Eastern Anatolian regions had only primary school degrees.

**Table 1:** Summary of participant characteristics.

**Participant profile and recruitment setting**	N^*^ of participants	**Mean age (Range)**	**Mean BMI (Range)**	**Parity**	**Residence**	**Highest educational qualification obtained**	**Employment status**	**Marital status**
Clinical patients: symptomatic patients undergoing laparoscopic surgery from hospital gynecology clinics	*N* = 40	34.9 (20–54)	23.0 (17.6–28.6)	*N* = 12 Nulliparous *N* = 28 Parous	*N* = 21 Istanbul *N* = 19 Eastern Anatolian region (*N* =1 Bingol, *N* = 1 Erzincan, *N* = 12 Erzurum, *N* = 3 Igdir, *N* = 1 Karliova, *N* = 1 Mus)	*N* = 11 Primary-school N = 1 Secondary-school *N* = 9 High-school *N* = 17 Undergraduate *N* = 1 Postgraduate *N* = 1 N/A	*N* = 4 Student *N* = 20 Home-maker *N* = 16 employed	*N* = 30 Married *N* = 10 Single
General population: Turkish-speaking women from random households	*N* = 40	35.5 (18–55)	23.2 (16.5–39.2)	*N* = 16 Nulliparous *N* = 24 Parous	*N* = 14 Nicosia *N* = 17 Famagusta *N* = 3 Kyrenia *N* = 2 Iskele	*N* = 2 Primary-school *N* = 10 High-school *N* = 14 Undergraduate *N* = 14 Postgraduate	*N* = 6 Student *N* = 34 Employed	*N* = 21 Married *N* = 14 Single *N* = 5 Divorced

* *N, number*.

Although the majority of the participants found the questionnaire to be clear, all commented that the questionnaire was too long. Forty percent of the participants commented on the length after completing Section D, with the rest commenting after completing the whole questionnaire. Despite skip patterns being included in the questionnaire, they were missed by 10% of the participants. As a result of this, we made the skip patterns more visually distinct on the questionnaire and recommend that participants be notified about the skip patterns before the questionnaire is started. Twenty percent of all participants with primary or secondary education commented that the following questions were repetitive: pelvic pain questions (Section C2), during or after vaginal intercourse (Section C15), and pelvic pain in general (Section C27). The research assistants explained the differences between the sections, and we saw that participants had not properly read and understood the instructions at the beginning of these subsections. As a result of this, we made the instructions more visually distinct and recommend instructing the participants to read the instructions in these subsections of the questionnaire before starting. One participant from Northern Cyprus with primary school level education had difficulties in understanding some of the medical terms (“pelvic” and “anxiety”), so these were clarified by introducing a text that described the terms in parentheses next to these terminologies (e.g., pelvic—the lower part of the abdomen and/or groin, and anxiety—a worry).

The format of the items on the pregnancy/fertility history questionnaire table (Section B1) was found to be complicated and difficult to understand by 5% of the participants at first. We revised the table to fit onto a single page and made the subquestions bold to counteract this. Two and a half percent of the participants stated that they found the format of questions E1 and E2 complicated, and so similar formatting changes were made to help with the understanding and interpretation of these questions. We recommend that participants be given the opportunity to contact the research assistants on the study to ask any questions they may have before they submit their completed questionnaire. Although the presence of a research assistant when the participants were completing their questionnaire was shown to be motivational, it is important that the research assistants are fully trained so that responses to participant questions are consistent in wording, detail, and tone, not to bias the answers to be given by the participants.

At the end of Section F, we originally asked participants to provide their contact details (name, phone number, email, and address) if they wanted to be informed of future studies. However, as this then made questionnaire identifiable, we removed this section and participants were able to provide their contact information voluntarily on the consent form, which we then stored in a separate database on high-compliance servers.

## Discussion

The aim of this study was to provide a protocol for the cross-cultural translation and adaptation of the WERF EPHect EPQ-M. Using the Turkish language as an example, we used cognitive testing on 80 participants who found the questionnaire to be informative and feasible. Despite the participants commenting on the length of the questionnaire, all were able to fully complete it in 30–60 min and could not point out which section/questions they felt could be eliminated. Although the level of detail that the questionnaire asks is high, we believe that capturing this information is essential for the successful characterization of endometriosis as well as important exposures such as pain symptomatology, menstrual and reproductive history, medical history, hormone use, infertility, and demographic and lifestyle information. To help with the successful completion of the questionnaire, we made the skip patterns more visible using formatting techniques and recommend that participants be informed about skip patterns to avoid completion of unnecessary questions. In addition to this, using an electronic online platform to administer the questionnaire could mean that questions that need to be skipped could be done so automatically, depending on the prior responses of participants to certain questions.

A limitation of this study is that we only cross-culturally adapted and translated the minimal version of the WERF EPHect questionnaire (EPQ-M), which excludes questions on symptoms or characteristics pertaining across the life course. Hence, these additional questions in the standard (EPHect EPQ-S) questionnaire would also need to be cross-culturally adapted and translated into Turkish in subsequent studies.

### Recommendations

Although the regions that we have collected data from (Turkey and Northern Cyprus) use the same wording of the Turkish language, Turkish-speaking Cypriots have a different dialect. Regardless, all participants were able to understand and complete the Turkish EPHect EPQ-M. The education level of the participants was an important factor in the participant's understanding of the medical terminology included in the questionnaire, and we clarified this by using more common terms. In addition to this, we found that participants with lower education levels appeared to pay less attention to the instructions at the start of the questionnaire, so we recommend that research assistants be available to participants so that they can ask any questions before completing the questionnaire. In order to avoid data breaches, contact information from participants should be collected on a separate form to the questionnaire.

As the physical and mental state of participants has been shown to affect the responses of participants in symptom-based questionnaires like the EPHect EPQ-M (9), we recommend that the Short Form Health Status Survey (SF-36v2) (10) or the Endometriosis Health Profile Questionnaire (EHP-30) (11) or both be administered prior to the administration of the EPHect EPQ-M in order to capture the health-related quality of life of participants. We did not include them in our study here because it requires each study to be individually registered to use them. In addition to this, depression and anxiety scales, such as the Beck Depression Inventory (BDI) (12, 13), the State Trait Anxiety Inventory (STAI) (14, 15) and/or the Hospital Anxiety and Depression Scale (HADS) (Aydemir, 1997; Zigmond and Snaith, 1983) can also be useful for stratification of participants.

This is the first paper that has successfully cross-culturally adapted and translated the WERF EPHect EPQ-M for content and face validity and should act as a tool to allow for further studies that wish to use this questionnaire in other languages. Standardized tools like these are essential in order to facilitate collaboration and aid in the design and conduction of global studies to ultimately expand our knowledge into understanding the mechanisms of endometriosis and help those affected.

### Afterword

The Turkish cross-culturally translated and adapted questionnaire is freely available in the Supplementary Material of this paper. However, we request that researchers who wish to use it cite both the original EPHect questionnaire paper as well as this paper along with any modifications they make in order to adapt it to their population.

## Data Availability Statement

The original contributions presented in the study are included in the article/Supplementary Material, further inquiries can be directed to the corresponding author/s.

## Ethics Statement

The studies involving human participants were reviewed and approved by (1) Oxford Tropical Research Ethics Committee (OxTREC) of the University of Oxford (OxTREC reference: 37-17). (2) Ethics Committee of Eastern Mediterranean University (ETK00-2017-0240). (3) Cerrahpasa University Faculty of Medicine Ethics Group (52825153-604.01-01-20831). The patients/participants provided their written informed consent to participate in this study.

## Author Contributions

CM, GK, GS, BY, PY, and NR: recruitment and cognitive testing. CM, GK, BS, MH, and NR: manuscript preparation. MH and NR: substantial contributions to conception and design of the study. BS, BY, PY, GS, BT, LH, SM, CB, KZ, UI, EO, MH, and NR: critically revised the manuscript. All authors read, contributed to, and approved the final manuscript.

## Conflict of Interest

CB reports grants from Bayer AG, other from AbbVie Inc, grants from Volition Rx, grants from MDNA Life Sciences, grants from Roche Diagnostics Inc, non-financial support from Population Diagnostics Ltd, other from ObsEva, and other from Flo Health, outside the submitted work. KZ reports grants from Bayer Healthcare, other from AbbVie Ltd, non-financial support from AbbVie Ltd, grants from MDNA Life Sciences, grants from Roche Diagnostics Inc, grants from Volition Rx, grants from Evotec (Lab282-Partnership programme with Oxford University), outside the submitted work; and board member (Secretary) of the World Endometriosis Society; Research Advisory Board member of Well-being of Women, UK (research charity); Chair, Research Directions Working Group, World Endometriosis Society. The remaining authors declare that the research was conducted in the absence of any commercial or financial relationships that could be construed as a potential conflict of interest.

## Supplementary Material

Supplementary Material Turkish EPHect EPQ-M.
