# Supplementary material for: Protocol for the Cultural Translation and Adaptation of the World Endometriosis Research Foundation Endometriosis Phenome and Biobanking Harmonization Project Endometriosis Participant Questionnaire (EPHect)
Source: Front Glob Womens Health. 2021 Mar 26;2:644609. doi: 10.3389/fgwh.2021.644609 (PMC8593963; doi:10.3389/fgwh.2021.644609)
Supplement: Supplementary Material — Turkish EPHect EPQ-M. [file Data_Sheet_1.DOCX]

# **WERF EPHect Anketi – Türkçe Minimum Versiyon (Turkish EPHect EPQ-M)**

# **Adet Geçmişi ve Hormonlar**

# **A1**. İlk defa adet gördüğünüzde kaç yaşındaydınız?

| 🞎 8 | 🞎 11 | 🞎 14 | 🞎 17’den yaşlı |
| --- | --- | --- | --- |
| 🞎 9 | 🞎 12 | 🞎 15 | 🞎 Belli değil |
| 🞎 10 | 🞎 13 | 🞎 16 | 🞎 Henüz olmadı, **lütfen C1 sorusuna geçiniz** |

# **A2**. **Son 3 ayda** hiç adet gördünüz mü? (*Tampon veya pede ihtiyaç duyduğunuz kanamaları kastediyoruz, sadece iç çamaşırınızda lekelenmeye neden* *olan akıntıları değil*)

| 🞎 Hayır **→ A2.1 ile devam ediniz** |  |
| --- | --- |

🞎 Evet → **A2.4’e geçiniz**

**Son 3 ayda hiç adet görmediyseniz:**

**A2.1**. Adet görmemenizin sebebi neydi?

🞎 Devamlı alınan hormonlar (Ör. hap, iğne, Mirena**,** HRT gibi)

🞎 Hamilelik/emzirme

🞎 Emin değilim

🞎 Diğer (*Lütfen açıklayın*) _____________________________________________________

**A2.2.** **Son 12 ayda** yaklaşık olarak kaç kez adet gördünüz? _____

## **A2.3.** Son adet döneminiz ne zamandı?

🞎 3-6 ay once 🞎 7-12 ay önce 🞎 12 aydan once

## **→Lütfen şimdi A3 sorusuyla devam edin.**

**Eğer son 3 ay adet gördüyseniz, lütfen son adet dönemlerinizle ilgili aşağıdaki sorulara cevap veriniz.**

**A2.4.** **Son 3 aydaki** adetleriniz kendiliğinden mi, yoksa hormon etkisiyle miydi (*Ör. hap, iğne, Mirena ya da HRT etkisiyle gibi)*?

| 🞎 Kendiliğinden | 🞎 Hormon etkisiyle |
| --- | --- |

## **A2.5.** Son adet döneminizin ilk günü hangi tarihti?

| Son adet dönemi _ _ /_ _ /_ _ _ _  GG AA YYYY | 🞎 Hatırlamıyorum |
| --- | --- |

## **A2.6.** **Son 3 ayda** adetleriniz düzenli miydi?

🞎 Aşırı düzenli (Beklenenden 1-2 gün önce veya sonra başlıyordu)

🞎 Çok düzenli (Beklenenden 3-4 gün önce veya sonra başlıyordu)

🞎 Düzenli (Beklenenden 5-7 gün önce veya sonra başlıyordu)

🞎 Biraz düzensiz (Beklenenden 8-20 gün önce veya sonra başlıyordu)

🞎 Düzensiz (Beklenenden 20 günden daha öncesi veya sonrası başlıyordu)

**A2.7. Son 3 aydaki** adet dönemlerinizde genellikle kaç gün kanamanız oluyordu? (*Sadece iç çamaşırınızda lekelenmeye neden olan akıntıları hesaba katmayın*)

_____ gün ya da 🞎 kesin bir şey söylemek için çok düzensizdi

**A2.8.** Aşağıdaki şekil adet döneminizde her 4 saatte bir gerçekleşebilecek kanama miktarlarının örneklerini gösteriyor. Lütfen **en yoğun** ve **ortalama geçirdiğiniz** adet dönemlerinizdeki dört saatlik kanamayı belirtiniz.

| **En ağır geçirdiğinizde?**  🞎Lekelenme  🞎Hafif  🞎Orta  🞎Ağır  **Ortalama geçirdiğinizde?**  🞎Lekelenme  🞎Hafif  🞎Orta  🞎Ağır |  |
| --- | --- |

**A2.9.** **Son üç ayda**, bir döneminizin ilk günü ile diğer döneminizin ilk günü arasında **ortalama** kaç gün vardı? *(Lekelenmeler hariç)*

🞎 24 günden az

🞎 24-31 gün arası

🞎 32-38 gün arası

🞎 39-50 gün arası

🞎 51 günden fazla

🞎 Düzensiz olduğu için birşey söyleyemiyorum

**A3.** Lütfen herhangi bir nedenden dolayı (akne, kramp, düzensiz adetler, gebelik kontrolü, gebelik tedavileri) aşağıdaki hormonlardan kullandıklarınızı listeleyin. Lütfen, kullanılan her hormon için, ne tür hormon kullanıldığını aşağıdaki kategorilerde belirtilen numaraları yazmak suretiyle belirtin. Lütfen ilk kez hangi yaşta kullandığınızı ve ne kadar süre kullandığınızı da belirtin. Eğer kullandığınız hormonun adını hatırlayamıyorsanız ilgili kutucuğa “bilmiyorum” yazın.

1=Doğum kontrol hapıyla birlikte (örneğin; Myralon, Yasmin, Microgynon)

2=Sadece progesterone içeren doğum kontrol hapı (“mini-hap”, örneğin; Cerazette, Desirette)

3=Hangi oral doğum kontrol hapı emin değilim

4=Progestin enjeksiyonu/yükleme (örneğin; Depo provera)

5=Transdermal’ler: flaster (örneğin; Climara),

6=Vajinal halka (NuvaRing)

7=Progesterone içeren RIA (Mirena)

8=Hormonal implant (Implanon)

9=Dönemleri düzenlemek için oral progestinler (örneğin; medroxyprogesteron asetat [Tarlusal/Farlutal], dİdrogesteron [Duphaston], dienogest [Visanne], Norethisterone)

10=GnRH Agonist/yükleme (örneğin; leuprolid (leuproline) acetate [Lucrin], goserelin [Zoladex])

11=Noretindron asetat (Primolut N)

12=Danazol (lütfen vajinal ya da oral olduğunu belirtiniz)

13=Hormon yerine koyma tedavisi

14=Diğer

15=Ne tür hormon olduğunu bilmiyorum

| Hormon Adı | Hormon Türü  (Lütfen yukarıdaki kategorilerdeki ilgili numarayı yazınız) | Başlangıç Yaşı | Son 3 ay içerisinde kullandınız mı? | Toplam kullandığınız süre | Eğer kullandığınız hormon enjeksiyon ise, lütfen son enjeksiyon tarihini yazınız |
| --- | --- | --- | --- | --- | --- |
| *Örneğin:*  *Yasmin* | *1* | *18* | ***✓*** *Hayır*  🞎 *Evet* | *…… ay*   1. *yıl* | *......./......./.......*  *Gün Ay Yıl* |
| 1. ............................................ | ........ | ........ | 🞎 Hayır  🞎 Evet | …… ay  …… yıl | *......./......./.......*  *Gün Ay Yıl* |
| 2. ............................................ | ........ | ........ | 🞎 Hayır  🞎 Evet | …… ay  …… yıl | *......./......./.......*  *Gün Ay Yıl* |
| 3. ............................................ | ........ | ........ | 🞎 Hayır  🞎 Evet | …… ay  …… yıl | *......./......./.......*  *Gün Ay Yıl* |
| 4. ............................................ | ........ | ........ | 🞎 Hayır  🞎 Evet | …… ay  …… yıl | *......./......./.......*  *Gün Ay Yıl* |
| 5. ............................................ | ........ | ........ | 🞎 Hayır  🞎 Evet | …… ay  …… yıl | *......./......./.......*  *Gün Ay Yıl* |
| 6. ............................................ | ........ | ........ | 🞎 Hayır  🞎 Evet | …… ay  …… yıl | *......./......./.......*  *Gün Ay Yıl* |
| 7. ............................................ | ........ | ........ | 🞎 Hayır  🞎 Evet | …… ay  …… yıl | *......./......./.......*  *Gün Ay Yıl* |
| 8. ............................................ | ........ | ........ | 🞎 Hayır  🞎 Evet | …… ay  …… yıl | *......./......./.......*  *Gün Ay Yıl* |

# **A4.** Hiç acil gebelik kontrolü (Ertesi gün hapları) kullandınız mı?

| 🞎 Hayır | 🞎 Evet |
| --- | --- |

**Eğer evet ise: A4.1.** **Son 3 ay** içerisinde acil gebelik kontrolü (Ertesi gün hapları) kullandınız mı?

🞎 Hayır

🞎 Evet

**A5.**  Hormonları kullanma sebepleriniz nedir/neydi? (*Lütfen tüm ilgili olanları ✓ şeklinde işaretleyin*)

🞎 Doğum kontrolü / gebelikten korunma

🞎 Pelvik (kasık) ağrısı ya da adet dönemlerinde ağrılar

## **Eğer evet ise: A5.1.** Hormonlar ağrıyı azalttı mı? 🞎 Hayır 🞎 Evet

## **A5.2.** Ağrıları azaltmada yetersiz olduğu için hiç hormonları kestiniz mi ya da hormon değiştirdiniz mi? 🞎 Hayır 🞎 Evet

🞎 Düzensiz adet dönemleri

🞎 Ağır adet dönemleri

🞎 Akne

🞎 Polikistik Over Sendromu (PKOS)

🞎 Yumurtalık kisti🞎 Diğer (*Lütfen belirtiniz*): __________________________________________

# **A6.** Hiç hormonal olmayan Rahimiçi araç (RİA, spiral) kullandınız mı?

| 🞎 Hayır | 🞎 Evet |
| --- | --- |

## **Eğer evet ise**: **A6.1** **İlk hangi yaşta** hormonal olmayan RİA kullandınız? _____

**A6.2 Son 3** ay içerisinde hormonal olmayan RİA kullandınız mı? 🞎 Evet 🞎 Hayır

**A6.3** **Ne kadar süre** hormonal olmayan RİA kullandınız? ____ay ____yıl

# **Gebelik ve Doğurganlık**

# **B1**. Hiç hamile kaldınız mı (Gebelik testi ile doğrulanan düşük, dış gebelik veya erken doğum)?

🞎 Hayır

🞎 Evet, lütfen aşağıdaki tabloyu doldurunuz.

|  | **Hamilelik Sırası** | | | | | | |
| --- | --- | --- | --- | --- | --- | --- | --- |
|  | **1.** | **2.** | **3.** | **4.** | **5.** | **6.** | **7.** |
| **Hamilelik başlangıcında kaç yaşındaydınız?** | | | | | | | |
| (Lütfen her hamilelikteki yaşınızı yazınız) | ____ | ____ | ____ | ____ | ____ | ____ | ____ |
| **Bu gebelik için -eğer varsa- hangi tedavi uygulandı?** | | | | | | | |
| Doğal gebelik: gebelik tedavisi yok | 🞎 | 🞎 | 🞎 | 🞎 | 🞎 | 🞎 | 🞎 |
| Haplarla yumurtlamayı uyaran gebelik ilaçları (Clomid, clomiphene) | 🞎 | 🞎 | 🞎 | 🞎 | 🞎 | 🞎 | 🞎 |
| İntrauterin döllenme (IUI) | 🞎 | 🞎 | 🞎 | 🞎 | 🞎 | 🞎 | 🞎 |
| Tüp bebek döllenme (IVF/ICSI) | 🞎 | 🞎 | 🞎 | 🞎 | 🞎 | 🞎 | 🞎 |
| **Bu gebeliğin sonucu ne oldu?** *(Lütfen tüm geçerli olanları ✓ şeklinde işaretleyin)* | | | | | | | |
| Tek bebek | 🞎 | 🞎 | 🞎 | 🞎 | 🞎 | 🞎 | 🞎 |
| İkiz ya da üçüz | 🞎 | 🞎 | 🞎 | 🞎 | 🞎 | 🞎 | 🞎 |
| Düşük | 🞎 | 🞎 | 🞎 | 🞎 | 🞎 | 🞎 | 🞎 |
| Ölü doğum | 🞎 | 🞎 | 🞎 | 🞎 | 🞎 | 🞎 | 🞎 |
| Kürtaj | 🞎 | 🞎 | 🞎 | 🞎 | 🞎 | 🞎 | 🞎 |
| Tubal ya da rahim dışında herhangi bir yerde gebelik  (Dış gebelik) | 🞎 | 🞎 | 🞎 | 🞎 | 🞎 | 🞎 | 🞎 |
| Mol gebeliği (Üzüm gebeliği) | 🞎 | 🞎 | 🞎 | 🞎 | 🞎 | 🞎 | 🞎 |
| Şu anda hamileyim | 🞎 | 🞎 | 🞎 | 🞎 | 🞎 | 🞎 | 🞎 |
| **Eğer bu gebelik düşük/dış gebelik ile sonuçlandıysa veya gebeliği sonlandırdıysanız, bu süreç nasıl yönetildi?** | | | | | | | |
| Kürtaj ile (D&C) | 🞎 | 🞎 | 🞎 | 🞎 | 🞎 | 🞎 | 🞎 |
| İlaçla (Tabletlerle, oral ya da vajinal) | 🞎 | 🞎 | 🞎 | 🞎 | 🞎 | 🞎 | 🞎 |
| Herhangi bir uygulamaya gerek yoktu | 🞎 | 🞎 | 🞎 | 🞎 | 🞎 | 🞎 | 🞎 |
| **Bu gebelik doğumla sonuçlandıysa, doğum vajinal yoksa sezaryan doğum muydu?** | | | | | | | |
| Vajinal doğum | 🞎 | 🞎 | 🞎 | 🞎 | 🞎 | 🞎 | 🞎 |
| Sezaryan doğum | 🞎 | 🞎 | 🞎 | 🞎 | 🞎 | 🞎 | 🞎 |
| **Doğumunuz kendiliğinden mi başladı?** | | | | | | | |
| Doğum yok | 🞎 | 🞎 | 🞎 | 🞎 | 🞎 | 🞎 | 🞎 |
| Spontan | 🞎 | 🞎 | 🞎 | 🞎 | 🞎 | 🞎 | 🞎 |
| Uyarılarak | 🞎 | 🞎 | 🞎 | 🞎 | 🞎 | 🞎 | 🞎 |
| **Gebelik ya da emzirmeye bağlı olarak aşağıdaki komplikasyonlardan birini yaşadınız mı?** | | | | | | | |
| Gebeliğe bağlı diyabet | 🞎 | 🞎 | 🞎 | 🞎 | 🞎 | 🞎 | 🞎 |
| Gebeliğe bağlı yüksek tansiyon | 🞎 | 🞎 | 🞎 | 🞎 | 🞎 | 🞎 | 🞎 |
| Preeklampsisi/ gebelik zehirlenmesi | 🞎 | 🞎 | 🞎 | 🞎 | 🞎 | 🞎 | 🞎 |
| Mastit/meme enfeksiyonu | 🞎 | 🞎 | 🞎 | 🞎 | 🞎 | 🞎 | 🞎 |
| HELLP sendromu | 🞎 | 🞎 | 🞎 | 🞎 | 🞎 | 🞎 | 🞎 |
| Hiperemezis gravidarum/ Gebelikte aşırı bulantı-kusma | 🞎 | 🞎 | 🞎 | 🞎 | 🞎 | 🞎 | 🞎 |
| Erken doğum (37 hafta öncesi doğum) | 🞎 | 🞎 | 🞎 | 🞎 | 🞎 | 🞎 | 🞎 |

| Diğer: ________________________ | 🞎 | 🞎 | 🞎 | 🞎 | 🞎 | 🞎 | 🞎 |
| --- | --- | --- | --- | --- | --- | --- | --- |
| Diğer: ________________________ | 🞎 | 🞎 | 🞎 | 🞎 | 🞎 | 🞎 | 🞎 |
| **Eğer bu gebelik doğumla sonuçlandıysa, ne kadar süre emzirdiniz?** | | | | | | | |
| (Lütfen emzirdiğiniz ay sayısını yazınız ya da emzirmediyseniz ‘0’ yazınız; 1 aydan az emzirdiyseniz, ‘1’ yazınız) | ____ | ____ | ____ | ____ | ____ | ____ | ____ |

**B2.** Olumsuz şekilde sonuçlanmak üzere hiç 6 aydan daha fazla süre hamile kalmaya çalıştınız mı?

🞎 Hayır

## 🞎 Evet **→** **Eğer evet ise:** **B2.1.** Hamile kalmayı denediğiniz en uzun süre ne kadardı? _____ ay

# **B3.** Size ya da eşinize, neden hamile kalmadığınızla ilgili test yapıldı mı/ araştırıldı mı?

🞎 Hayır

🞎 Evet **→** **Eğer evet ise: B3.1.** Bu testlerin sonucu ne idi? *(Lütfen tüm geçerli olanları işaretleyin ✓)*

| 🞎 Endometriozis  (Çikolata kisti) | 🞎 Pelvik inflamatuar hastalık | 🞎 Hiç sebep bulunamadı |
| --- | --- | --- |
| 🞎 Yapışıklık | 🞎 Hiç/düzensiz yumurtlama | 🞎 Hatırlamıyorum |
| 🞎 Tıkalı tüpler | 🞎 Zayıf sperm miktarı/kalitesi | 🞎 Diğer: _____________ |
| 🞎 Polikistik over sendromu | 🞎 Miyomlar |  |

# **B4.** Herhangi bir klinikte gebelik için hiç tedavi arayışına girdiniz mi?

🞎 Hayır

🞎 Evet **→** **Eğer evet ise: B4.1.** Lütfen denediğiniz tedavi hakkında bilgi veriniz.

|  | **Hiç denemedim** | **Son 3 ayda denedim** | **Denedim fakat son 3 ay içerisinde değil** | **Denediğiniz dönem sayısı (eğer varsa)** |
| --- | --- | --- | --- | --- |
| Planlanmış cinsel ilişki | 🞎 | 🞎 | 🞎 | ____ |
| Gebe kalmaya odaklı cinsel ilişki | 🞎 | 🞎 | 🞎 | ____ |
| Ağızdan alınan haplar ile yumurta gelişiminin tetiklenmesi (Klomid, klomifen veya hap formunda herhangi bir ilaç) | 🞎 | 🞎 | 🞎 | ____ |
| Enjeksiyon yoluyla alınan ilaçlar (Gonadotropinler, HCG veya enjeksiyon yoluyla alınan başka bir ilaç) | 🞎 | 🞎 | 🞎 | ____ |
| Progesteron (Vajinal veya intramusküler enjeksiyon) | 🞎 | 🞎 | 🞎 | ____ |
| Eşin/Partner ‘in semeni ile rahim içi inseminasyon (IUI) | 🞎 | 🞎 | 🞎 | ____ |
| Donör spermi ile rahim içi inseminasyon (IUI) | 🞎 | 🞎 | 🞎 | ____ |
| In vitro fertilizasyon (IVF) | 🞎 | 🞎 | 🞎 | ____ |
| Intrasitoplazmik sperm enjeksiyonu (ICSI) (Mikroenjeksiyon) ile in vitro fertilizasyon | 🞎 | 🞎 | 🞎 | ____ |
| Yumurtaların donörden alınarak gerçekleştirildiği in vitro fertilizasyon | 🞎 | 🞎 | 🞎 | ____ |

**B4.2.** Eğer **IVF, ICSI** (Mikroenjeksiyon) veya **Donör yumurta ile IVF** tedavisi geçirdiyseniz: Tedavi siklusunuz/sikluslarınız aşağıdaki hangi aşamada sonlandı? *(Lütfen uygun olan tüm seçenekleri* ***✓*** *şeklinde işaretleyin)*

🞎 Yumurta büyütme aşaması (Yumurta elde edilemedi)

🞎 Yumurta toplama aşaması (Transfer edilecek embriyo oluşmadı)

🞎 Embriyo transferi (Pozitif gebelik sonucu alınamadı)

🞎 Kimyasal gebelik (Pozitif gebelik sonucu sonrası ultrasonografide kalp atışı gözlenmedi)

🞎 Klinik gebelik (Kalp atımı görüldü, fakat 12 haftadan önce gebelik kaybı yaşandı)

🞎 12 haftadan sonra gebelik kaybı veya ölü doğum

🞎 Canlı doğum

# **Ağrı**

**C1.** Herkes hayatının bir bölümünde ağrılı durumlarla karşılaşır. Bu durumlar baş ağrısı, diş ağrısı, eklem ağrısı veya kas ağrısı olabilir. Bunun yanında kişiler sıklıkla hastalık, yaralanma, dişle ilgili operasyonlar veya cerrahi sonucu da ağrıya maruz kalırlar.

Bu tabloda ağrınız olduğunda ne hissettiğiniz ve düşündüğünüzle ilgileniyoruz. Aşağıda ağrıyla ilişkili olabilecek farklı duygu ve düşünceleri tanımlayan 13 cümle bulunmaktadır. Lütfen aşağıdaki ölçeği kullanarak ağrınız olduğunda hissettiğiniz düşüncelerinizi ve duygularınızı derecelendiriniz.

|  | **Hiç** | **Biraz** | **Orta derecede** | **Ciddi Şekilde** | **Her zaman** |
| --- | --- | --- | --- | --- | --- |
| Ağrının bitip bitmeyeceği hakkında sürekli endişelenirim. | 🞎 | 🞎 | 🞎 | 🞎 | 🞎 |
| Bu duruma daha fazla katlanamayacağımı hissederim. | 🞎 | 🞎 | 🞎 | 🞎 | 🞎 |
| Bu ağrının korkunç olduğunu ve hiçbir zaman iyileşmeyeceğini düşünürüm. | 🞎 | 🞎 | 🞎 | 🞎 | 🞎 |
| Bu ağrının berbat olduğunu ve beni mahvettiğini hissederim. | 🞎 | 🞎 | 🞎 | 🞎 | 🞎 |
| Buna daha fazla dayanamayacağımı hissederim. | 🞎 | 🞎 | 🞎 | 🞎 | 🞎 |
| Ağrı daha da kötüleşecek diye korkarım. | 🞎 | 🞎 | 🞎 | 🞎 | 🞎 |
| Başka ağrılı durumları düşünüp dururum. | 🞎 | 🞎 | 🞎 | 🞎 | 🞎 |
| Tedirgin bir şekilde ağrının geçmesini isterim. | 🞎 | 🞎 | 🞎 | 🞎 | 🞎 |
| Ağrıyı aklımdan bir türlü çıkaramadığımı fark ederim. | 🞎 | 🞎 | 🞎 | 🞎 | 🞎 |
| Sürekli ağrının ne kadar acı verdiğini düşünürüm. | 🞎 | 🞎 | 🞎 | 🞎 | 🞎 |
| Ağrının durmasını ne kadar çok istediğimi düşünürüm. | 🞎 | 🞎 | 🞎 | 🞎 | 🞎 |
| Ağrının şiddetini azaltmak için yapabileceğim hiçbir şeyin olmadığını düşünürüm. | 🞎 | 🞎 | 🞎 | 🞎 | 🞎 |
| Ciddi bir durumun ortaya çıkıp çıkmayacağını merak ederim. | 🞎 | 🞎 | 🞎 | 🞎 | 🞎 |

| **Aşağıdaki sorular adet dönemlerinizdeki pelvik (Kasık ve alt karın) ağrı hakkındadır (*Düzensiz kanama veya hormonlu tedavi alt*ı*nda ki kanamalar dahil*).**  'Pelvik (Kasık ve alt karın) ağrı' ile, karnınızın alt kısmındaki, bu resimdeki gölgeli alan tarafından gösterildiği gibi, her tür ağrıyı kastediyoruz (Kramp, çekilme, bıçaklanma vb.): |  |
| --- | --- |

# **C2.** Adet dönemlerinizde pelvik (Kasık ve altkarın) ağrı hissettiğiniz zamanlar oldu mu?

🞎 Hayır olmadı

🞎 Hafif kramplar (Tedavi hiç gerekmedi ya da çok nadiren gerekti)

🞎 Orta düzeyde kramplar (Tedavi genellikle gerekti)

🞎 Şiddetli kramplar (Tedavi ve yatak istirahatı gerekti)

## **C2.1.** Hangi yaşta adet dönemi ağrılarınız başladı?____yaşında

# **Son 3 ay içerisinde adet gördüyseniz, lütfen aşağıdaki soruları cevaplayınız, aksi takdirde burayı işaretleyiniz ____ ve C12 sorusundan devam ediniz.**

## **C3. Son adet döneminizde** ne kadar ağrı çektiniz?

🞎 Ağrı yoktu **→ C9'a geçiniz**

🞎 Hafif kramplar (Tedavi hiç gerekmedi ya da çok nadiren gerekti)

🞎 Orta düzeyde kramplar (Tedavi genellikle gerekti)

🞎 Şiddetli kramplar (Tedavi ve yatak istirahatı gerekti)

### **C4. Son adet döneminizde** pelvik ağrı için hiç ağrı kesici aldınız mı? (*Lütfen ilgilileri işaretleyiniz)*

🞎 Hayır

🞎 Evet, doktor tarafından verilen ağrı kesici

🞎 Evet, ama reçetesiz aldığım ağrı kesici (Ör. parasetamol, majezik, apranax, nurofen [ibuprofen], minoset)

### **C5. Son adet döneminizde** pelvik ağrıların hafifletilmesine yardımcı olmak için hormon ilaçları aldınız mı? Öyleyse ağrınızı azaltmaya yardımcı oldu mu?

🞎 Hayır, ağrı için hormon almadım

🞎 Evet, ama ağrılar azalmadı

🞎 Evet, ağrılar azaldı

### **C6. Son adet döneminizde,** pelvik ağrılarınız işe, okula gitmenize engel oldu mu ya da günlük işlerinizi yapmanıza engel oldu mu (Ağrı kesici alsanız dahi)?

🞎 Hayır 🞎 Evet

### **C7. Son adet döneminizde,** pelvik ağrılarınızdan dolayı günün herhangi bir bölümü içinde uzanmanız gerekti mi?

### 🞎 Hayır 🞎 Evet

# **C8. Son adet döneminizde** pelvik ağrılarınız en şiddetli haldeyken ağrılarınızın şiddetini ölçekte belirtiniz.

# *(0=ağrı yok ve 10=akla gelebilecek en şiddetli ağrı durumlarını ifade ediyor.)*

**Ağrı yok**

**Aşırı ağrılı**

|  | |  |  |  |  |  |  |  | | |
| --- | --- | --- | --- | --- | --- | --- | --- | --- | --- | --- |
| 0 | 1 | 2 | 3 | 4 | 5 | 6 | 7 | 8 | 9 | 10 |

# **C9. Son 12 ay içerisinde**, adet dönemlerinizde ne kadar sıklıkta pelvik ağrı çektiniz?

🞎 Hiç

🞎 Ara sıra (Gördüğüm adetlerin 4'te birinden az)

🞎 Sık sık (Gördüğüm adetlerin 4'te birinde ya da yarısında)

🞎 Genellikle (Gördüğüm adetlerin yarısından fazlasında)

🞎 Her zaman (Her adet dönemimde)

## **C10. Son 12 ay içerisinde**, pelvik ağrılarınız en şiddetli haldeyken ağrılarınızın şiddetini ölçekte belirtiniz.

**Ağrı yok**

**Aşırı ağrılı**

## *(0=ağrı yok ve 10=akla gelebilecek en şiddetli ağrı durumlarını ifade ediyor.)*

|  | |  |  |  |  |  |  |  | | |
| --- | --- | --- | --- | --- | --- | --- | --- | --- | --- | --- |
| 0 | 1 | 2 | 3 | 4 | 5 | 6 | 7 | 8 | 9 | 10 |

### **C11.** Aşağıdaki sorular **son 3 aydaki adet dönemlerinizde** pelvik ağrı çektiğinizde, bağırsak hareketleriniz/dışkınızla ilgilidir.

| **Son 3 ay içerisinde adet dönemlerinizde pelvik ağrınız olduğu zamanlarda, ne kadar sık...** | **Hiç/ Nadiren** | **Bazen** | **Sık sık** | **Çoğu zaman** | **Her zaman** |
| --- | --- | --- | --- | --- | --- |
| (a) …bağırsak hareketlerinizden sonra, ağrılarınız azaldı ya da bitti mi? | 🞎 | 🞎 | 🞎 | 🞎 | 🞎 |
| (b) …bağırsak hareketlerinizden sonra, ağrılarınız daha kötü oldu mu? | 🞎 | 🞎 | 🞎 | 🞎 | 🞎 |
| (c) …pelvik ağrılarınız başladığında daha sık bağırsak hareketleriniz oldu mu? | 🞎 | 🞎 | 🞎 | 🞎 | 🞎 |
| (d) …pelvik ağrılarınız başladığında daha seyrek bağırsak hareketleriniz oldu mu? | 🞎 | 🞎 | 🞎 | 🞎 | 🞎 |
| (e) …ağrılarınız başladığında dışkılarınız daha yumuşak mıydı? | 🞎 | 🞎 | 🞎 | 🞎 | 🞎 |
| (f) …ağrılarınız başladığında dışkılarınız daha sert miydi? | 🞎 | 🞎 | 🞎 | 🞎 | 🞎 |

**Aşağıdaki sorular hayatınızda adet dönemlerinizde pelvik ağrılarınızın en kötü olduğu zamanlarla ilgilidir.**

# **C12.** Adet dönemlerinizde en ağır pelvik ağrıları çektiğinizde kaç yaşındaydınız? _____ yaşında

## **C13.** En ağrılı pelvik ağrısı çektiğiniz adet döneminizdeki ağrı şiddetini lütfen 0'dan 10'a sayılar kullanarak anlatın. *(0=ağrı yok ve 10=akla gelebilecek en şiddetli ağrı durumlarını ifade ediyor.)*

**Ağrı yok**

**Aşırı ağrılı**

|  | |  |  |  |  |  |  |  | | |
| --- | --- | --- | --- | --- | --- | --- | --- | --- | --- | --- |
| 0 | 1 | 2 | 3 | 4 | 5 | 6 | 7 | 8 | 9 | 10 |

## **C14.** En ağrılı pelvik ağrısı çektiğiniz adet döneminizde, ağrıları hafifletmek için hiç ağrı kesici/hormon alıyor muydunuz?

*(Lütfen tüm ilgili olanları ✓ şeklinde işaretleyin)*

🞎 Hayır

🞎 Evet, doktor tarafından verilen ağrı kesiciler aldım

🞎 Evet, reçetesiz aldığım ağrı kesiciler kullandım (Ör. parasetamol, majezik, apranax, nurofen [ibuprofen], minoset)

🞎 Evet, hormon aldım, fakat ağrıları azaltmadı

🞎 Evet, hormon aldım, ve ağrılar azaldı

| **Aşağıdaki sorular vajinal ilişki sırasında veya sonrasında pelvik (Kasık ve alt karın) ağrı ile ilgilidir.**    'Pelvik ağrı' ile, karnınızın alt kısmındaki, bu resimdeki gölgeli alan tarafından gösterildiği gibi, her tür ağrıyı kastediyoruz (Kramp, çekilme, bıçaklanma vb.): |  |
| --- | --- |

***Verdiğiniz tüm bilgilerin gizli tutulacağını hatırlatmak isteriz.***

# ***Bu sorulara cevap vermek istemiyorsanız, lütfen burayı işaretleyin ___ ve C27 sorusuna geçiniz.***

# ***Hiç cinsel ilişkiye girmediyseniz****,* ***lütfen burayı işaretleyiniz ___ ve C27 sorusuna geçiniz.***

# **C15.** Hiç cinsel ilişki sırasında veya vajinal cinsel ilişki sonrasında 24 saat içinde pelvik ağrılarınız oldu mu?

🞎 Hayır **→** **C27'ye geçiniz**

🞎 Evet **→** **Eğer evet ise:** **C15.1.** Bu ağrı hangi yaşta başladı? _____

## **C16.** En son ne zaman vajinal cinsel ilişkiniz oldu?

🞎 Son bir ayda

🞎 1-3 ay önce

🞎 4-12 ay önce

🞎 12 aydan önce **→** Öyleyse, **C16.1.** Pelvik ağrı yüzünden cinsel ilişkiden kaçındınız mı?

🞎 Hayır 🞎 Evet

**Eğer vajinal cinsel ilişkiniz 12 aydan daha önceyse lütfen C25'ye geçiniz.**

# **C17.** Son vajinal cinsel ilişki yaşadığınız sırada veya **24 saat** içinde pelvik ağrınız oldu mu?

🞎 Hayır **→ C25'a geçiniz.**

🞎 Evet, cinsel ilişki sırasında

🞎 Evet, cinsel ilişki sonrasında 24 saat içinde

🞎 Evet, hem cinsel ilişki sırasında hem de takip eden 24 saat içinde

**C18.** En son vajinal ilişki yaşadığınızda, ağrıyı nerede hissettiniz? (*Lütfen tüm ilgili olanları ✓ şeklinde işaretleyin*)

🞎 Vajina girişinde

🞎 Vajina içlerinde

🞎 Karında/pelvik bölgede

🞎 Başka yerde **→** **Eğer evet ise:** **C18.1** Lütfen belirtiniz:_______________________________

## **C19.** Lütfen son vajinal ilişkinizde pelvik ağrılarınızın en yoğun olduğu durumdaki şiddetini ölçekte belirtiniz.

## *(0=ağrı yok ve 10=akla gelebilecek en şiddetli ağrı durumlarını ifade ediyor.)*

**Ağrı yok**

**Aşırı ağrılı**

|  | |  |  |  |  |  |  |  | | |
| --- | --- | --- | --- | --- | --- | --- | --- | --- | --- | --- |
| 0 | 1 | 2 | 3 | 4 | 5 | 6 | 7 | 8 | 9 | 10 |

## **C20.** Lütfen son vajinal ilişkinizden sonraki 24 saat içerisindeki pelvik ağrılarınızın en yoğun olduğu durumdaki şiddetini ölçekte belirtiniz. *(0=ağrı yok ve 10=akla gelebilecek en şiddetli ağrı durumlarını ifade ediyor.)*

**Ağrı yok**

**Aşırı ağrılı**

|  | |  |  |  |  |  |  |  | | |
| --- | --- | --- | --- | --- | --- | --- | --- | --- | --- | --- |
| 0 | 1 | 2 | 3 | 4 | 5 | 6 | 7 | 8 | 9 | 10 |

## **C21. Son 12 aydaki** vajinal ilişki yaşadığınız zamanlarda ilişki sırasında veya sonrasındaki 24 saat içinde ne sıklıkta pelvik ağrılarınız oldu?

🞎 Hiç

🞎 Ara sıra (Dörtte birinden azında)

🞎 Genellikle (Dörtte biriyle yarısı arasında)

🞎 Genellikle (Yarısından fazlasında)

🞎 Her zaman (Her seferinde)

**C22. Son 12 ayda**, vajinal ilişkinizin diğerlerinden daha ağrılı olduğu bir dönem var mıydı? (*Lütfen tüm ilgili olanları ✓ şeklinde işaretleyin*)

|  | İlgili dönemde vajinal ilişki oldu mu? |  | **Evet ise:** Diğerlerinden daha ağrılı mıydı? |
| --- | --- | --- | --- |
| Adet dönemi sürecinde? | 🞎 Hayır 🞎 Evet | 🡪 | 🞎 Hayır 🞎 Evet |
| Adet döneminden birkaç gün önce | 🞎 Hayır 🞎 Evet | 🡪 | 🞎 Hayır 🞎 Evet |
| Adet döneminden birkaç gün sonra | 🞎 Hayır 🞎 Evet | 🡪 | 🞎 Hayır 🞎 Evet |
| Adet dönemlerinin arasında (Yumurtlama dönemlerinde) | 🞎 Hayır 🞎 Evet | 🡪 | 🞎 Hayır 🞎 Evet |

## **C23. Son 12 ayda**, pelvik ağrınız nedeniyle vajinal ilişki **kesintiye** uğradı mı?

🞎 Hayır 🞎 Evet

## **C24. Son 12 ayda**, pelvik ağrınız nedeniyle vajinal ilişkiden **kaçındınız** mı?

🞎 Hayır 🞎 Evet

**Aşağıdaki sorular, hayatınızdaki vajinal ilişki sırasında yaşadığınız en yoğun ağrılı dönemlerle ilgilidir.**

# **C25.** Vajinal ilişki sırasında en yoğun pelvik ağrısı yaşadığınızda kaç yaşındaydınız? ______ yaşında

# **C26.** Vajinal ilişki sırasında en yoğun pelvik ağrısı yaşadığınızda ağrılarınızın şiddetini ölçekte belirtiniz.

# *(0=ağrı yok ve 10=akla gelebilecek en şiddetli ağrı durumlarını ifade ediyor.)*

**Ağrı yok**

**Aşırı ağrılı**

| 0 | 1 | 2 | 3 | 4 | 5 | 6 | 7 | 8 | 9 | 10 |
| --- | --- | --- | --- | --- | --- | --- | --- | --- | --- | --- |

| **Aşağıdaki sorular, genel olarak pelvik (Kasık ve alt karın) ağrı hakkındadır.** 'Pelvik (Kasık ve alt karın) ağrı' ile, karnınızın alt kısmındaki, bu resimdeki gölgeli alan tarafından gösterildiği gibi, her tür ağrıyı kastediyoruz (Kramp, çekilme, bıçaklanma vb.): |  |
| --- | --- |

# **C27.** Hiç genel olarak pelvik (Kasık ve alt karın) ağrı yaşadınız mı? **Şunları saymayın**: *menstrüel kramplara, ilişkiye, ameliyata, gebeliğe, doğuma, sporla ilgili veya diğer yaralanmalara, gıda zehirlenmesine veya mide üşütmesine bağlı ağrılar.*

🞎 Hayır **→** **C40'a geçiniz**

🞎 Evet **→ Eğer evet ise: C27.1** Hangi yaşta pelvik ağrınız başladı? ___ yaşında

**C27.2** En son ne zaman bu ağrınız oldu?

🞎 Geçen ay

🞎 1-3 ay önce

🞎 4-6 ay önce

**Lütfen C36'ya geçiniz**

🞎 7-12 ay önce

🞎 12 aydan eski

### **C28.** Ağrınız, **son 3 ayda** aşağıdaki faaliyetlerinizi ne derece etkiledi?

|  | **Hiç** | **Biraz** | **Orta** | **Epey** | **Aşırı** | **Bilmiyorum** |
| --- | --- | --- | --- | --- | --- | --- |
| İş veya okul: | 🞎 | 🞎 | 🞎 | 🞎 | 🞎 | 🞎 |
| Evdeki günlük aktiviteler: | 🞎 | 🞎 | 🞎 | 🞎 | 🞎 | 🞎 |
| Uyku: | 🞎 | 🞎 | 🞎 | 🞎 | 🞎 | 🞎 |
| Cinsel ilişki: | 🞎 | 🞎 | 🞎 | 🞎 | 🞎 | 🞎 |
| Egzersiz/spor: | 🞎 | 🞎 | 🞎 | 🞎 | 🞎 | 🞎 |
| Sosyal aktiviteler: | 🞎 | 🞎 | 🞎 | 🞎 | 🞎 | 🞎 |

### **C29.** Tahminen **son 3 ayda** toplamda ne kadar bu ağrıyı çektiniz?

| 🞎 Ayda bir günden az  🞎 Ayda bir gün  🞎 Ayda iki üç gün | 🞎 Haftada bir gün  🞎 Haftada bir günden fazla  🞎 Her gün |
| --- | --- |

### **C30. Son 3 ayda** ağrınızı azaltmak için hiç tedavi gördünüz mü? (*Lütfen tüm olanları ✓ şeklinde işaretleyiniz)*

🞎 Hayır

🞎 Evet, doktorun verdiği ağrı kesici

🞎 Evet, ama reçetesiz ağrı kesici (Ör. parasetamol, majezik, apranax, nurofen [ibuprofen],

minoset)

🞎 Evet, hormon ama ağrıyı azaltmadı

🞎 Evet, hormon, ağrıyı azalttı

**C31.** Lütfen **son 3 ayda** ağrınız **en kötü** haldeyken ağrınızın şiddetini ölçek ile belirtiniz.

*(0=ağrı yok ve 10=akla gelebilecek en şiddetli ağrı durumlarını ifade ediyor.)*

| **Ağrı yok**  **Aşırı ağrılı** | |  |  |  |  |  |  |  | | |
| --- | --- | --- | --- | --- | --- | --- | --- | --- | --- | --- |
| 0 | 1 | 2 | 3 | 4 | 5 | 6 | 7 | 8 | 9 | 10 |

### **C32. Son 3 ayda** pelvik ağrınız olduğunda, ağrı nasıldı?

|  | **Yoktu** | **Hafif** | **Orta** | **Şiddetli** |
| --- | --- | --- | --- | --- |
| Zonklama | 🞎 | 🞎 | 🞎 | 🞎 |
| Çekilme | 🞎 | 🞎 | 🞎 | 🞎 |
| Bıçak saplanır gibi | 🞎 | 🞎 | 🞎 | 🞎 |
| Keskin | 🞎 | 🞎 | 🞎 | 🞎 |
| Kramp | 🞎 | 🞎 | 🞎 | 🞎 |
| Kemirici | 🞎 | 🞎 | 🞎 | 🞎 |
| Yanıcı | 🞎 | 🞎 | 🞎 | 🞎 |
| Ağrıma | 🞎 | 🞎 | 🞎 | 🞎 |
| Ağır | 🞎 | 🞎 | 🞎 | 🞎 |
| Gerici | 🞎 | 🞎 | 🞎 | 🞎 |
| Parçalayıcı | 🞎 | 🞎 | 🞎 | 🞎 |
| Yorucu-Tüketici | 🞎 | 🞎 | 🞎 | 🞎 |
| Hasta edici | 🞎 | 🞎 | 🞎 | 🞎 |
| Korkunç | 🞎 | 🞎 | 🞎 | 🞎 |
| Eziyet edici | 🞎 | 🞎 | 🞎 | 🞎 |

### **C33.** Pelvik ağrınızı ne daha kötü yapar? (*Lütfen tüm geçerli olanları ✓ şeklinde işaretleyin).*

| 🞎 Oturmak | 🞎 Cinsel ilişki ya da orgasm | 🞎 Hava |
| --- | --- | --- |
| 🞎 idrar yapma hissi ve idrar yapmak | 🞎 Egzersiz | 🞎 Giyinmeme bağlı |
| 🞎 Bağırsak hareketleri | 🞎 Stres | 🞎 Öksürme/hapşırma |
| 🞎 Kabızlık | 🞎 Belirli saatlerde | 🞎 Etkileyen bir şey yok |
| 🞎 Ayakta durmak ya da yürümek | 🞎 Midem doluyken | 🞎 Diğer (*Lütfen belirtiniz*):__________ |

### **C34.** Pelvik ağrınızı ne hafifletir? (*Lütfen tüm geçerli olanları ✓ şeklinde işaretleyin*).

| 🞎 Ağrı kesici | 🞎 Buz | 🞎 Laksatif / enema |
| --- | --- | --- |
| 🞎 Rahatlama | 🞎 Isıtma pedi | 🞎 İdrar yapmak |
| 🞎 Uzanma | 🞎 Bağırsak hareketleri | 🞎 TENS |
| 🞎 Müzik | 🞎 Sıcak duş | 🞎 Etkileyen bir şey yok |
| 🞎 Masaj | 🞎 Meditasyon | 🞎 Diğer (*Lütfen belirtiniz*): ___________________________ |

### **C35.** Aşağıdaki sorular **son 3 aydaki** pelvik ağrılarınız sırasında bağırsak hareketleriniz/dışkınız hakkındadır.

| **Son 3 ayda** **pelvik ağrınız olduğunda, Ne kadar sık...** | **Hiç/**  **Nadiren** | **Bazen** | **Sık sık** | **Çoğu zaman** | **Her zaman** |
| --- | --- | --- | --- | --- | --- |
| (a) …bağırsak hareketlerinizden sonra ağrınız daha iyi oldu mu ya da durdu mu? | 🞎 | 🞎 | 🞎 | 🞎 | 🞎 |
| (b) …bağırsak hareketlerinizden sonra ağrınız daha kötüye gitti mi? | 🞎 | 🞎 | 🞎 | 🞎 | 🞎 |
| (c) …ağrınız olduğunda daha sık bağırsak hareketleriniz oldu mu? | 🞎 | 🞎 | 🞎 | 🞎 | 🞎 |
| (d) …ağrınız olduğunda daha seyrek bağırsak hareketleriniz oldu mu? | 🞎 | 🞎 | 🞎 | 🞎 | 🞎 |
| (e) …ağrınız olduğunda dışkınız daha yumuşak mıydı? | 🞎 | 🞎 | 🞎 | 🞎 | 🞎 |
| (f) …ağrınız olduğunda dışkınız daha sert miydi? | 🞎 | 🞎 | 🞎 | 🞎 | 🞎 |

**Aşağıdaki sorular hayatınızdaki pelvik (Kasık/alt karın) ağrınızın en kötü olduğu zamanlarla ilgilidir.** *Lütfen şunları saymayın: adet veya cinsel ilişkiye, hamileliğe veya doğuma, herhangi bir ameliyata, sporla ilgili veya diğer yaralanmalara, gıda zehirlenmesine veya mide üşütmesine bağlı ağrılar.*

### **C36.** Pelvik (Kasık/alt karın) ağrınız **en kötü** durumdayken kaç yaşındaydınız? _____ yaşında

#### **C37.** Lütfen pelvik (Kasık/alt karın) ağrınız **en kötü** durumdayken ağrınızın şiddetini ölçek ile belirtiniz.

*(0=ağrı yok ve 10=akla gelebilecek en şiddetli ağrı durumlarını ifade ediyor.)*

**Ağrı yok**

**Aşırı ağrılı**

|  | |  |  |  |  |  |  |  | | |
| --- | --- | --- | --- | --- | --- | --- | --- | --- | --- | --- |
| 0 | 1 | 2 | 3 | 4 | 5 | 6 | 7 | 8 | 9 | 10 |

#### **C38.** Hayatınızda pelvik (Kasık/alt karın) ağrınız **en kötü** durumdayken ağrılarınızın azalması için tedavi gördünüz mü? *(Lütfen tüm ilgili olanları ✓ şeklinde işaretleyin)*

🞎 Hayır

🞎 Evet, doktorun verdiği ağrı kesici

🞎 Evet, reçetesiz aldığım ağrı kesici (Ör. parasetamol, majezik, apranax, ibuprofen, minoset)

🞎 Evet, hormon, fakat ağrıyı hafifletmedi

🞎 Evet, hormon, ağrıyı hafifletti

**C39.** Ağrınıza bir doktordan tarafından teşhis konuldu mu?

🞎 Hayır

🞎 Evet **→ Eğer evet ise:** *(Lütfen tüm ilgili olanları ✓ şeklinde işaretleyin)*

🞎 İrritabl bağırsak sendromu

🞎 İnflamatuar bağırsak hastalığı (Ör. Crohn hastalığı veya Ülseratif kolit)

🞎 Endometriozis (Çikolata kisti)

🞎 Miyomlar

🞎 Yumurtalık kisti

🞎 Pelvik inflamatuar hastalık / enfeksiyon

🞎 Ağrılı mesane / interstisyel sistit (Bakteriyel mesane enfeksiyonu **değil**)

🞎 Stres

🞎 Diğer (*Lütfen açıklayınız*): ________________________

# **C40.** Lütfen **son 12 ay içinde** aşağıdaki diğer ağrı türlerini yaşayıp yaşamadığınızı belirtin:

|  | **Hayır** | **Evet, geçen ay** | **Evet, 1 aydan daha önce** |
| --- | --- | --- | --- |
| Bel ağrısı | 🞎 | 🞎 | 🞎 |
| Viral enfeksiyon veya (Spor) yaralanma ile ilgisi olmayan kas/eklem ağrısı | 🞎 | 🞎 | 🞎 |
| Yumurtlamada ağrı | 🞎 | 🞎 | 🞎 |
| Bacaklarda ağrı | 🞎 | 🞎 | 🞎 |
| İdrar yollarında ağrı | 🞎 | 🞎 | 🞎 |
| Bağırsak hareketi ile ağrı | 🞎 | 🞎 | 🞎 |

# **Tıbbi Geçmiş**

# **D1.** Daha önce bir doktor tarafından size hiç kanser teşhisi konuldu mu?

🞎 Hayır

🞎 Evet 🡪 **Eğer evet ise: D1.1.** Ne tür kanser(ler) teşhisi konuldu (Birincil yer) ve ilk tanısı ne zaman

konuldu? (*Lütfen aşağıda belirtiniz.)*

| **Kanser türü** | **Tanı konulduğunda yaşınız** |
| --- | --- |
|  |  |
|  |  |
|  |  |

**D2.** Şimdiye kadar aşağıdaki tıbbi durumlardan herhangi biri sizde saptandı mı? Evet ise, ilk kez hangi yaşta bir doktor tarafından teşhis konuldu? (*Lütfen tüm ilgili olanları ✓ şeklinde işaretleyin*)

| **Tıbbi Durum** | | | **Teşhis Yaşı** | **Tıbbi Durum** | | | **Teşhis Yaşı** |
| --- | --- | --- | --- | --- | --- | --- | --- |
| **Hayır** | **Evet** |  |  | **Hayır** | **Evet** |  |  |
| 🞎 | 🞎 | İlaç ya da terapi gerektiren anksiyete |  | 🞎 | 🞎 | İrritabl bağırsak sendromu (IBS) |  |
| 🞎 | 🞎 | Astım |  | 🞎 | 🞎 | Migren |  |
| 🞎 | 🞎 | Kalp damar hastalığı |  | 🞎 | 🞎 | Mitral kapak prolapsus |  |
| 🞎 | 🞎 | Crohn hastalığı |  | 🞎 | 🞎 | Multipl skleroz |  |
| 🞎 | 🞎 | Kronik yorgunluk sendromu (CFS) /  Miyaljik ensefalomiyelit (ME) |  | 🞎 | 🞎 | Ağrılı mesane / interstisyel sistit (bakteriyel mesane enfeksiyonu DEĞİL) |  |
| 🞎 | 🞎 | Sağırlık/duyma zorluğu |  | 🞎 | 🞎 | Pelvik Inflamatuar Hastalık (PID) |  |
| 🞎 | 🞎 | İlaç ya da terapi gerektiren depresyon |  | 🞎 | 🞎 | Polikistik Over Sendromu |  |
| 🞎 | 🞎 | Kontrol gerektiren diyabet |  | 🞎 | 🞎 | Romatoid artrit |  |
| 🞎 | 🞎 | Insülin ya da ilaç gerektiren diyabet |  | 🞎 | 🞎 | Skolyoz (omurganın eğriliği) |  |
| 🞎 | 🞎 | Egzema |  | 🞎 | 🞎 | Omurga sorunları (skolyoz hariç) |  |
| 🞎 | 🞎 | Miyomlar |  | 🞎 | 🞎 | Sjogren sendromu-göz kuruluğu |  |
| 🞎 | 🞎 | Fibromiyalji |  | 🞎 | 🞎 | SLE (Lupus) |  |
| 🞎 | 🞎 | Glandular ateş |  | 🞎 | 🞎 | Tiroid hastalığı |  |
| 🞎 | 🞎 | Graves hastalığı |  | 🞎 | 🞎 | Ülseratif kolit |  |
| 🞎 | 🞎 | Hashimoto hastalığı |  | 🞎 | 🞎 | Diğer (*Lütfen belirtin*): |  |
| 🞎 | 🞎 | Yüksek tansiyon |  |  |  | _______________________________ |  |

# **D3.** Rahim, rahim ağzınız veya vajinanızda yapısal bir sorun/konjenital (doğumsal) sorun olduğu söylendi mi?

🞎 Hayır

🞎 Evet 🡪 **Eğer evet ise: D3.1.** Bununla ilgili ameliyat oldunuz mu?

🞎 Hayır

🞎 Evet 🡪 **Eğer evet ise: D3.2.** Ameliyat sonrasında sorun hafifledi ya- da bitti mi? 🞎 Hayır

🞎 Evet

# **D4.** Hayatınız boyunca aşağıdaki cerrahi prosedürlerden birini yaşadınız mı? Eğer öyleyse, yaklaşık olarak hangi yaş(lar)da prosedür(ler)i yaşadınız, toplamda kaçı vardı ve ameliyatın nedeni neydi?

| **Cerrahi işlemler** | **Hayır** | **Evet** | **Evetse:** | | |
| --- | --- | --- | --- | --- | --- |
|  |  |  | **Toplamda kaç kez?** | **Yaş(lar)ınız** | **Ameliyat sebebi neydi?** |
| Tüp ligasyonu (sterilizasyon / tüpleri bağlama) | 🞎 | 🞎 |  | Yaş: …….. |  |
| Appendektomi | 🞎 | 🞎 |  | Yaş: …….. |  |
| Histerektomi | 🞎 | 🞎 |  | Yaş: …….. |  |
| Ooforektomi  Evet ise, yumurtalıklarınızın kaç tanesi alındı?  🞎 1 🞎 ikisi de 🞎 emin değilim | 🞎 | 🞎 |  | Yaş(lar): ………...  ..................... |  |
| Kürtaj (Dilatasyon ve Küretaj, D & C) | 🞎 | 🞎 |  | Yaş(lar): ………...  ..................... |  |
| Rahim boynu cerrahi (LEEP veya konizasyon) | 🞎 | 🞎 |  | Yaş(lar): ………...  ..................... |  |
| Histeroskopi | 🞎 | 🞎 |  | Yaş(lar): ………...  ..................... |  |
| Safra kesesi ameliyatı | 🞎 | 🞎 |  | Yaş(lar): ………...  ..................... |  |
| Fıtık ameliyatı | 🞎 | 🞎 |  | Yaş(lar): ………...  ..................... |  |
| Sigmoidoskopi / kolonoskopi (bağırsağınıza bakmak için bir tüp yerleştirilmesi) | 🞎 | 🞎 |  | Yaş(lar): ………...  ..................... |  |
| Laparoskopi (karın içine bir teleskop yerleştirilmesi ile ilgili ameliyat) | 🞎 | 🞎 |  |  |  |
| 1^inci^ |  |  |  | Yaş: …….. |  |
| 2^inci^ |  |  |  | Yaş: …….. |  |
| 3^üncü^ |  |  |  | Yaş: …….. |  |
| 4^üncü^ |  |  |  | Yaş: …….. |  |
| 5^inci^ ya da sonuncu |  |  |  | Yaş: …….. |  |
| Diğer karın ameliyatları: ……………………………………………………. | 🞎 | 🞎 |  | Yaş(lar): ………...  ..................... |  |

# **D5.** Aşağıdaki sorular genel olarak **son 3 aydaki** bağırsak hareketleriniz / dışkınızla ilgilidir**:**

| **Son 3 ayda, ne kadar sık...** | **Hiçbir zaman /Nadiren** | **Bazen** | **Sık sık** | **Çoğu zaman** | **Her zaman** |
| --- | --- | --- | --- | --- | --- |
| …sulu dışkınız oldu mu? | 🞎 | 🞎 | 🞎 | 🞎 | 🞎 |
| …sert dışkınız oldu mu? | 🞎 | 🞎 | 🞎 | 🞎 | 🞎 |

# **D6.** **Son 3 ayda** aşağıdaki durumlarla karşılaştınız mı? *(Lütfen tüm uygun olanları ✓ şeklinde işaretleyin)*

| 🞎 Rektal kanama ya da dışkıda kan | 🞎 Bağırsak hareketleri sırasında zorlanma |
| --- | --- |
| 🞎 Haftada 3 defadan az bağırsak hareketleri | 🞎 Acil tuvalet ihtiyacı |
| 🞎 Günde 3 defadan fazla bağırsak hareketleri | 🞎 Bağırsaklarınızın tam boşalamaması hissi |
| 🞎 Mide bulantısı ve/ya da kusma | 🞎 Tuvalet sırasında dışkıda mucus |
| 🞎 Bağırsak krampları | 🞎 Karın şişliği |

# **D7.** **Son 3 ayda**, aşağıdakileri yaşadınız mı? *(Lütfen tüm uygun olanları ✓ şeklinde işaretleyin)*

| 🞎 Öksürürken, gülerken, hapşırırken idrar kaçırma | 🞎 İdrar sonrası çok kısa süre içinde tekrar idrar yapmak |
| --- | --- |
| 🞎 İdrar yaparken zorlanma | 🞎 İdrar yapmaya rağmen tekrar idrar yapma ihtiyacı |
| 🞎 Sık sık mesane enfeksiyonları | 🞎 İdrarda kan |

# **D8.** Doktor tarafından size endometriozis (Çikolata kisti) teşhisi konuldu mu?

# 🞎 Hayır

🞎 Evet 🡪 **D8.1 ile devam ediniz**

**D8.1.** Teşhis nasıl dokunuldu? *(Lütfen tüm uygun olanları ✓ şeklinde işaretleyin)*

🞎 Laparoskopi ya da diğer cerrahi işlem

🞎 Ultrason/MR

🞎 Semptomplara bağlı olarak

🞎 Diğer (*Lütfen belirtiniz*): ___________________________________________

## **D8.2.** Eğer endometriozis ameliyatı olduysanız, son ameliyat sonrasında iyileştiniz mi?

🞎 Hayır

🞎 Evet

🞎 Emin değilim

🞎 Endometriozis için ameliyat olmadım

## **D8.3.** İlk semptomları yaşadığınızda kaç yaşındaydınız? _____ yaşında

**veya** semptom yoksa burayı işaretleyin **🞎**

## **D8.4.** Endometriozis tanısı konulmadan önce, eğer semptom varsaydı, hangi semptomlar doktora gitmenize neden oldu? *(Lütfen tüm geçerli olanları ✓ şeklinde işaretleyin)*

🞎 Ağrı

🞎 Kısırlık

🞎 Semptom yoktu

🞎 Diğer (*Lütfen belirtiniz*): _________________________________________

## **D8.5.** Endometriozis teşhisi konulduğunda kaç yaşındaydınız? _____ yaşında

# **D9.** Endometriozis tespiti için hiç ameliyat olup da bir şey bulunmadığı oldu mu?

🞎 Hayır

🞎 Evet 🡪 **Eğer evet ise: D9.1.** Hangi semptomlar sizi ameliyata itti?

🞎 Ağrı

🞎 Kısırlık

🞎 Diğer (*Lütfen belirtiniz*): ___________________________________

# **D10.** Kanbağı olan akrabalarınıza aşağıdaki hastalıklardan hangilerinin teşhisi konuldu?

| Durum | Anne | Kız kardeş | Anne tarafından, büyükanne, teyze ya da kuzen | Baba tarafından büyükanne, hala ya da kuzen |
| --- | --- | --- | --- | --- |
| Endometriozis | 🞎 Hayır  🞎 Evet  🞎 Bilmiyorum | 🞎 Hayır  🞎 Evet  🞎 Bilmiyorum  🞎 Kız kardeşim yok | 🞎 Hayır  🞎 Evet  🞎 Bilmiyorum | 🞎 Hayır  🞎 Evet  🞎 Bilmiyorum |
| Kronik pelvik ağrı | 🞎 Hayır  🞎 Evet  🞎 Bilmiyorum | 🞎 Hayır  🞎 Evet  🞎 Bilmiyorum  🞎 Kız kardeşim yok | 🞎 Hayır  🞎 Evet  🞎 Bilmiyorum | 🞎 Hayır  🞎 Evet  🞎 Bilmiyorum |

# **İlaç Kullanımı**

**E1.** Lütfen 3 **ay ya da daha uzun süre,** haftada en az bir kere reçeteli veya reçetesiz ağrı kesici ilaç kullandıysanız belirtiniz.

**AĞRI KESİCİ İLAÇ TABLOSU**

| **İlaç tipi** | **Hiç**  **kullandınız**  **mı?**  **evet ise *✓*** | **Şu anda kullanıyor musunuz?**  **evet ise *✓*** | **İlk kez düzenli**  **kullanmaya başladığınızda**  **kaç yaşındaydınız?** | **Hangi ağrı**  **için**  **kullandınız?** | **Haftada kaç**  **gün kullandınız?** | **Haftada kaç**  **tablet kullandınız?** | **Toplamda ne**  **kadar süre**  **kullandınız?** |
| --- | --- | --- | --- | --- | --- | --- | --- |
| Parasetamol/  asetaminofen  (Ör. Minoset) | ­­  🞎 | ­­  🞎 | ____ | 🞎 Pelvik ağrı  🞎 Diğer ağrı  🞎 İkisi de | 🞎 1  🞎 2-3  🞎 4-5  🞎 6+ | 🞎 1-2  🞎 3-5  🞎 6-14  🞎 15+ | ____ ay  ____ yıl |
| Aspirin (325 mg veya  daha fazla/tablet) | ­­  🞎 | ­­  🞎 | ____ | 🞎 Pelvik ağrı  🞎 Diğer ağrı  🞎 İkisi de | 🞎 1  🞎 2-3  🞎 4-5  🞎 6+ | 🞎 1-2  🞎 3-5  🞎 6-14  🞎 15+ | ____ ay  ____ yıl |
| Ibuprofen (Ör. Brufen, nurofen) | ­­  🞎 | ­­  🞎 | ____ | 🞎 Pelvik ağrı  🞎 Diğer ağrı  🞎 İkisi de | 🞎 1  🞎 2-3  🞎 4-5  🞎 6+ | 🞎 1-2  🞎 3-5  🞎 6-14  🞎 15+ | ____ ay  ____ yıl |
| Celebrex | ­­  🞎 | ­­  🞎 | ____ | 🞎 Pelvik ağrı  🞎 Diğer ağrı  🞎 İkisi de | 🞎 1  🞎 2-3  🞎 4-5  🞎 6+ | 🞎 1-2  🞎 3-5  🞎 6-14  🞎 15+ | ____ ay  ____ yıl |
| Diğer anti-inflamatuar analjezikler (Ör. Kataflan, novalgin, nimes, majezik, apranax [naproxen], voltaren, minoset plus) | ­­  🞎 | ­­  🞎 | ____ | 🞎 Pelvik ağrı  🞎 Diğer ağrı  🞎 İkisi de | 🞎 1  🞎 2-3  🞎 4-5  🞎 6+ | 🞎 1-2  🞎 3-5  🞎 6-14  🞎 15+ | ____ ay  ____ yıl |
| Güçlü (narkotik) analjezikler (Ör. hydrocodone +parasetamol, codeine+parasetamol, morfin, codeine, oxycodone, hydrocodone, Demerol) | ­­  🞎 | ­­  🞎 | ____ | 🞎 Pelvik ağrı  🞎 Diğer ağrı  🞎 İkisi de | 🞎 1  🞎 2-3  🞎 4-5  🞎 6+ | 🞎 1-2  🞎 3-5  🞎 6-14  🞎 15+ | ____ ay  ____ yıl |
| Sinirleri/merkezi sinir sistemini etkileyen diğer ağrı kesiciler (Ör. Lirika, galara, amitriptyline, nortriptyline, gabapentin, pregabalin, lamotrigine) | ­­  🞎 | ­­  🞎 | ____ | 🞎 Pelvik ağrı  🞎 Diğer ağrı  🞎 İkisi de | 🞎 1  🞎 2-3  🞎 4-5  🞎 6+ | 🞎 1-2  🞎 3-5  🞎 6-14  🞎 15+ | ____ ay  ____ yıl |
| Kas gevşeticiler (Ör. diazepam/temazepam, buscopan, muscoril, muscoflex) | ­­  🞎 | ­­  🞎 | ____ | 🞎 Pelvik ağrı  🞎 Diğer ağrı  🞎 İkisi de | 🞎 1  🞎 2-3  🞎 4-5  🞎 6+ | 🞎 1-2  🞎 3-5  🞎 6-14  🞎 15+ | ____ ay  ____ yıl |
| Bitkisel ilaçlar (Ör.  Acı kırmızı biber türleri [Capsicum] Hayıt ağacı [Vitex agnus-castus L.] meyve ekstresi) | ­­  🞎 | ­­  🞎 | ____ | 🞎 Pelvik ağrı  🞎 Diğer ağrı  🞎 İkisi de | 🞎 1  🞎 2-3  🞎 4-5  🞎 6+ | 🞎 1-2  🞎 3-5  🞎 6-14  🞎 15+ | ____ ay  ____ yıl |

# **E2.** **3 aydan uzun süre** hiç reçeteli ilaç aldınız mı, hormon tedavileri ve ağrı ilaçları hariç?

# 🞎 Evet **→** Lütfen aşağıdaki **Reçeteli ilaç tablosunu doldurunuz**

🞎 Hayır **→** **F1’e geçiniz**

## **REÇETELİ İLAÇ TABLOSU**

| **İlaç tipi** | | **Bu ilacı hiç her gün olacak şekilde, bir aydan fazla aldınız mı?** | **İlk hangi yaşta her gün olacak şekilde bir aydan fazla aldınız?** | **Toplamda kaç yıl aldınız? Eğer bir yıldan az aldıysanız lütfen “0 yıl” kullanın.** | **Şu anda ilacı kullanıyor musunuz?** | **Lütfen biliyorsanız en güncel kullandığınız ilacın adını yazınız.** |
| --- | --- | --- | --- | --- | --- | --- |
|  |  | Evet ise*✓* | İlk yaş | Toplam yıl: | Evet ise*✓* | İlaç adı: |
| Diüretik (idrar söktürücü hap) | | 🞎 | __ __ | __ __ | 🞎 |  |
| Şeker ilacı | | 🞎 | __ __ | __ __ | 🞎 |  |
| Insulin | | 🞎 | __ __ | __ __ | 🞎 |  |
| Tiroid ilacı | | 🞎 | __ __ | __ __ | 🞎 |  |
| Epilepsi ilacı | | 🞎 | __ __ | __ __ | 🞎 |  |
| Uyku ilacı/sakinleştirici | | 🞎 | __ __ | __ __ | 🞎 |  |
| Anti-depresan | | 🞎 | __ __ | __ __ | 🞎 |  |
| Ruhsal problemler için diğer ilaçlar | | 🞎 | __ __ | __ __ | 🞎 |  |
| Osteoporoz ilacı (“ kemik erimesi”) | | 🞎 | __ __ | __ __ | 🞎 |  |
| Eklem iltihabı ilacı | | 🞎 | __ __ | __ __ | 🞎 |  |
| Bİr ay ya da daha fazla antibiyotik | | 🞎 | __ __ | __ __ | 🞎 |  |
| Antiasit (Mide yanması ilacı) | | 🞎 | __ __ | __ __ | 🞎 |  |
| Mide ülseri/ gastrit ilacı | | 🞎 | __ __ | __ __ | 🞎 |  |
| Yüksek kolesterol ilacı | | 🞎 | __ __ | __ __ | 🞎 |  |
| Alerji ilacı (Antihistamin) | | 🞎 | __ __ | __ __ | 🞎 |  |
| Steroid (Oral, inhaler, veya burundan) | 🞎 | __ __ | __ __ | 🞎 |  | |
| Kanser için kemoterapi | 🞎 | __ __ | __ __ | 🞎 |  | |
| Kanser için tamoksifen | 🞎 | __ __ | __ __ | 🞎 |  | |
| Tansiyon ilacı | 🞎 | __ __ | __ __ | 🞎 |  | |
| Anjina (Göğüs ağrısı) ilacı | 🞎 | __ __ | __ __ | 🞎 |  | |
| Kalp için diğer ilaçlar | 🞎 | __ __ | __ __ | 🞎 |  | |
| Astım için solunum cihazı | 🞎 | __ __ | __ __ | 🞎 |  | |
| Warfarin / heparin kan sulandırıcı | 🞎 | __ __ | __ __ | 🞎 |  | |
| Migren ilacı/iğnesi | 🞎 | __ __ | __ __ | 🞎 |  | |
| Diğer 1: ________________ | 🞎 | __ __ | __ __ | 🞎 |  | |
| Diğer 2: ________________ | 🞎 | __ __ | __ __ | 🞎 |  | |
| Diğer 3: ________________ | 🞎 | __ __ | __ __ | 🞎 |  | |

# **Kişisel Bilgiler Ve Yaşam Tarzı**

# **F1.** Doğum tarihiniz nedir? __ __ / __ __ / __ __ __ __

# **F2.** Okula gidiyor musunuz?

🞎 Hayır 🞎 Evet

# **F3**. Mezun olduğunuz en yüksek derece nedir?

🞎 İlkokul

🞎 Ortaokul

🞎 Lise

🞎 Önlisans

🞎 Lisans

🞎 Yüksek lisans/doktora

# **F4.** Şu andaki boyunuz nedir?

# _______ cm

# **F5.** Şu andaki kilonuz nedir?

# _______ kg

# **F6.** 18 yaşınızdayken doğal saç renginiz nasıldı? *(Lütfen 1 tane işaretleyin*).

| 🞎 Siyah | 🞎 Koyu kahverengi | 🞎 Açık kahverengi | 🞎 Sarışın | 🞎 Kızıl |
| --- | --- | --- | --- | --- |

# **F7.** Göz renginiz nedir? *(Lütfen 1 tane işaretleyin).*

| 🞎 Kahverengi | 🞎 Ela | 🞎 Yeşil | 🞎 Mavi | 🞎 Gri |
| --- | --- | --- | --- | --- |

**F8.** Son 12 ay içerisinde, aşağıdaki aktivitelere haftada ortalama kaç saat harcadınız?

|  | **Hiç** | **1-4 dk.** | **5-19 dk.** | **20-59 dk.** | **1 saat** | **1-1.5 saat** | **2-3 saat** | **4-6 saat** | **7-10 saat** | **11+ saat** |
| --- | --- | --- | --- | --- | --- | --- | --- | --- | --- | --- |
| Yürüyüş (İşe yürümek dahil) | 🞎 | 🞎 | 🞎 | 🞎 | 🞎 | 🞎 | 🞎 | 🞎 | 🞎 | 🞎 |
| Yavaş koşu (6km/saatten yavaş) | 🞎 | 🞎 | 🞎 | 🞎 | 🞎 | 🞎 | 🞎 | 🞎 | 🞎 | 🞎 |
| Koşu (6km/saatten hızlı) | 🞎 | 🞎 | 🞎 | 🞎 | 🞎 | 🞎 | 🞎 | 🞎 | 🞎 | 🞎 |
| Bisiklet (Koşu bisikleti dahil) | 🞎 | 🞎 | 🞎 | 🞎 | 🞎 | 🞎 | 🞎 | 🞎 | 🞎 | 🞎 |
| Jimnastik/aerobik/aerobik dans | 🞎 | 🞎 | 🞎 | 🞎 | 🞎 | 🞎 | 🞎 | 🞎 | 🞎 | 🞎 |
| Tenis, squash | 🞎 | 🞎 | 🞎 | 🞎 | 🞎 | 🞎 | 🞎 | 🞎 | 🞎 | 🞎 |
| Yüzme | 🞎 | 🞎 | 🞎 | 🞎 | 🞎 | 🞎 | 🞎 | 🞎 | 🞎 | 🞎 |
| Diğer aktiviteler: ______________ | 🞎 | 🞎 | 🞎 | 🞎 | 🞎 | 🞎 | 🞎 | 🞎 | 🞎 | 🞎 |

# **F9.** Hayatınız boyunca 100 sigaradan fazla içtiniz mi?

🞎 Hayır

## 🞎 Evet 🡪 **Eğer evet ise:** **F9.1.** İlk sigara içtiğinizde kaç yaşındaydınız? _______ yaşında

## **F9.2.** Halen sigara içiyor musunuz?

🞎 Hayır şu yaşımda bıraktım: ________

🞎 Evet ve haftada yaklaşık ________ sigara içiyorum

# **F10.** Alkol kullanıyor musunuz?

🞎 Hayır

🞎 Evet 🡪 **Eğer evet ise: F10.1** Ortalama bir haftada, aşağıdakilerden ne kadar içiyorsunuz?

(*Lütfen tam sayılar yazmaya çalışınız, 1-3 arası gibi ifadeler kullanmayınız*)

| **Alkol türü (miktar)** | **Haftada ne kadar tüketiyorsunuz?** |
| --- | --- |
| Bira/lager/cider (284 ml) | ______________ |
| Şarap (175 ml) | ______________ |
| Sert içki (25 ml) | ______________ |
| Diğer (*Lütfen belirtiniz*): _______________ | ______________ |

**F11.** Lütfen bu anketi bitirdiğiniz tarihi yazınız: __ __ / __ __ / __ __ __ __

Gün Ay Yıl

**Zamanınızı harcayıp katıldığınız için teşekkür ederiz. Yorum veya sorularınız varsa, buraya yazabilir veya bize katılımcı bilgilendirme formundaki telefon veya email adresinden ulaşabilirsiniz.**

_______________________________________________________________________________________

_______________________________________________________________________________________

_______________________________________________________________________________________
